# Post-Graduate Study in Scotland and Ireland

**Published:** 1908-09-05

**Authors:** 


### GRADUATE STUDY IN SCOTLAND AND IRELAND.

### IN SCOTLAND.

The four Scottish Universities have been but little distinguished in the past for their encouragement of post-graduate study. St. Andrews and Aberdeen concern themselves with the graduate no further than by offering him a degree in Public Health. Beyond this, Glasgow University offers courses in practical bacteriology, pathological histology, physiological chemistry, embryology, and a few of the special departments. Special courses are provided at Anderson's College Medical School and at St. Mungo's College. At the Glasgow Royal Infirmary the members of the hospital staff have now arranged an eighth series of post-graduate classes. These are to be opened on September 1. The staff of the Royal Infirmary have organised a comprehensive series of practical and clinical classes. The previous courses have already proved very successful, and have been well attended. Apply to Dr. Maxtone Thom, Superintendent of the Royal Infirmary.

In Edinburgh a more elaborate scheme of post-graduate study is available. The University of this city, in addition to teaching the subject of public health, has for some years provided advanced classes in the ordinary medical subjects for senior students and graduates, such as bacteriology, organic chemistry, and embryology, while numerous laboratories attached to the various professorial chairs have been open for research under certain restrictions. These laboratories are increasing yearly in equipment and efficiency, mainly as a result of the funds derived from Mr. Andrew Carnegie's gift of £2,000,000 to the Scottish Universities. For this purpose, too, a magnificent laboratory has been organised and equipped by the Royal College of Physicians of Edinburgh, where any medical graduate may obtain permission to conduct scientific research.

The University and the Royal Colleges of Edinburgh provide courses for graduates who have obtained their diploma—medical officers home on furlough, practitioners resident in country districts, foreign doctors anxious to study English, and any others who may desire a brief *resumé* of the essentials and recent advances in various special subjects.

The Post-Graduate Course extends this year from Monday, August 31, to Saturday, September 26. It comprises :—1. A General Course, the composite fee for which is five guineas for the four weeks, or three guineas for either the first or second fortnight 2. A Surgical Course, which includes the following classes : Operative surgery, surgical anatomy, surgical pathology, and *x*-rays applied to surgery, exclusively reserved for those attending the Surgical Course, the composite fee for which will be ten guineas. The entries for the Surgical Course will be limited to twenty-five. 3. Special classes on bacteriology, diseases of the blood, ear and throat, errors of refraction, practical gynæcology, and ophthalmoscopy, the fee for each being one guinea. These classes will be confined to graduates who have entered for the Surgical Course or for the General Course during the corresponding period. Full information and copies of the syllabus may be obtained from the Secretary, University New Buildings, Edinburgh.

### IN IRELAND.

A three weeks' post-graduates' course is given during the summer session at Trinity College, Dublin. The fee is £5 5s. A limited number of the class can live in the college rooms and dine in hall, at a charge of £1 per week. Apply Dr. Alfred Parsons, Lower Fitzwilliam Street, Dublin. A school has been formed to prepare candidates for the Royal Navy, the Royal Army Medical Corps, and the Indian Medical Service. The classes are held twice a year. Apply Dr. C. A. K. Ball, Merrion Square, Dublin.

At Belfast special summer classes are formed in bacteriology, clinical pathology, neurology, chemical physiology, and during the winter facilities are afforded to graduates to work at these subjects.

